# The Saturniidae of Barro Colorado Island, Panama: A model taxon for studying the long‐term effects of climate change?

**DOI:** 10.1002/ece3.3515

**Published:** 2017-10-22

**Authors:** Yves Basset, Greg P.A. Lamarre, Tom Ratz, Simon T. Segar, Thibaud Decaëns, Rodolphe Rougerie, Scott E. Miller, Filonila Perez, Ricardo Bobadilla, Yacksecari Lopez, José Alejandro Ramirez, Annette Aiello, Héctor Barrios

**Affiliations:** ^1^ Smithsonian Tropical Research Institute Ancon Panamá; ^2^ Faculty of Science University of South Bohemia Ceske Budejovice Czech Republic; ^3^ Maestria de Entomologia Universidad de Panamá Panama City Panama; ^4^ Institute of Entomology, Biology Centre Czech Academy of Science Ceske Budejovice Czech Republic; ^5^ Institute of Evolutionary Biology University of Edinburgh Edinburgh UK; ^6^ Centre d'Ecologie Fonctionnelle et Evolutive UMR 5175, CNRS–Université Montpellier–Univesrsité Paul‐Valéry–EPHE–SupAgroMontpellier–INRA–IRD Montpellier France; ^7^ Institut de Systématique Evolution, Biodiversité UMR 7205, CNRS–MNHN–UPMC–EPHE–Sorbonne Universités Paris France; ^8^ National Museum of Natural History Smithsonian Institution Washington DC USA

**Keywords:** climatic anomalies, DNA barcoding, functional groups, monitoring, population dynamics, rainforest, species traits

## Abstract

We have little knowledge of the response of invertebrate assemblages to climate change in tropical ecosystems, and few studies have compiled long‐term data on invertebrates from tropical rainforests. We provide an updated list of the 72 species of Saturniidae moths collected on Barro Colorado Island (BCI), Panama, during the period 1958‐2016. This list will serve as baseline data for assessing long‐term changes of saturniids on BCI in the future, as 81% of the species can be identified by their unique DNA Barcode Index Number, including four cryptic species not yet formally described. A local species pool of 60 + species breeding on BCI appears plausible, but more cryptic species may be discovered in the future. We use monitoring data obtained by light trapping to analyze recent population trends on BCI for saturniid species that were relatively common during 2009‐2016, a period representing >30 saturniid generations. The abundances of 11 species, of 14 tested, could be fitted to significant time‐series models. While the direction of change in abundance was uncertain for most species, two species showed a significant increase over time, and forecast models also suggested continuing increases for most species during 2017‐2018, as compared to the 2009 base year. Peaks in saturniid abundance were most conspicuous during El Niño and La Niña years. In addition to a species‐specific approach, we propose a reproducible functional classification based on five functional traits to analyze the responses of species sharing similar functional attributes in a fluctuating climate. Our results suggest that the abundances of larger body‐size species with good dispersal abilities may increase concomitantly with rising air temperature in the future, because short‐lived adults may allocate less time to increasing body temperature for flight, leaving more time available for searching for mating partners or suitable oviposition sites.

## 1 INTRODUCTION

Integrated monitoring of invertebrates, vertebrates, and plants may provide a complementary understanding of biodiversity changes in the face of global climate change (Thomas et al., 2004). Invertebrates are particularly sensitive to climate changes. For example, Thomas et al. (2004) reported that in Great Britain, the proportions of species declining in abundance over the last 20 years that can be directly attributed to climate change are 71%, 54%, and 28% for butterflies, birds, and vascular plants, respectively. Tropical insects often exhibit short generation times, as low as eight generations per year, with extremes to 10–14 generations for insect pests (Nair, 2007). Therefore, arthropod monitoring during 5–10 years in the tropics may involve 40–80 overlapping insect generations and may be particularly useful for developing early warning systems, but that idea remains to be tested rigorously for a variety of insect taxa.

Tropical insects may be extremely sensitive to local climate changes and anomalies for several reasons. First, the tropical regions, with their narrow annual temperature fluctuations, are thought to generate insect populations with higher and narrower thermal limits (Kaspari, Clay, Lucas, Yanoviak, & Kay, 2014). Projected air temperatures in the tropics approach the thermal limits of many insects over which muscular coordination may be disrupted or lost (Deutsch et al., 2008). Because insects are ectothermic organisms, they face limited tolerance to a warming world and hence hold greatest risks of extinction in the tropics (Deutsch et al., 2008; Kaspari et al., 2014; Singer & Parmesan, 2010). Second, tropical insects are very sensitive to desiccation and therefore to extreme drought or rainfall events (Hood & Tschinkel, 1990; Singer & Parmesan, 2010; Wolda, 1992). Finally, climate changes and anomalies may produce phenological mismatches between insect herbivores and plant resources, although this has been demonstrated mainly in temperate systems (Singer & Parmesan, 2010).

In general, we have little knowledge of the response of invertebrate assemblages to global and local climate change in tropical ecosystems, and very few studies have compiled long‐term data on invertebrates from tropical rainforests with notable exceptions of fruit‐feeding butterfly assemblages (Grøtan, Lande, Engen, Saether, & DeVries, 2012; Leidner, Haddad, & Lovejoy, 2010; Valtonen et al., 2013). Our knowledge is even scarcer regarding the response of tropical insects to climatic anomalies. For example, in Central America, sea surface temperatures, which characterize El Niño Southern Oscillation (ENSO) and La Niña events, are typically associated with more prolonged and extreme dry seasons (IPCC 2012; Lyon, 2004). These extreme climatic conditions such as El Niño influence the abundance of some butterfly species migrating across the Panama canal (Srygley, Dudley, Oliveira, & Riveros, 2014) and also can cause severe outbreaks of Lepidoptera (Van Bael et al., 2004).

In addition, species that share similar functional attributes (but not necessarily a common phylogeny) may have similar responses to climate fluctuations, and these responses may be more apparent and easy to interpret than when using a more traditional species‐specific approach (Pau et al., 2011). In temperate systems, functional categories have been used for Lepidoptera as relevant surrogates for life‐history changes induced by climatic shifts (Diamond, Frame, Martin, & Buckley, 2011; Slade et al., 2013). However, in the tropics, where typically taxonomic impediment is high and knowledge of life histories extremely limited, species traits often are difficult to measure and are limited to a few taxa, such as butterflies or ants (Parr et al., 2017; Xing et al., 2016).

**Figure 1:**
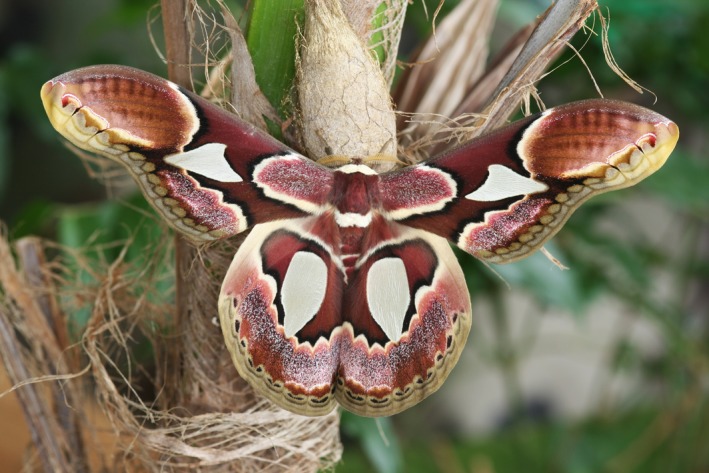
*Rothschildia erycina* (male), a species present on Barro Colorado Island, pictured near Cayenne, French Guiana, by Stéphane Brûlé

The Saturniidae (wild silk moths, Lepidoptera) are among the largest and most spectacular moths (Figure 1). The most recent assessment of their diversity reported 2,349 species in 169 genera worldwide (van Nieukerken et al., 2011), with most species occurring in the Neotropics (Scoble, 1995). Some of them are important agricultural pests (CAB International, 1998); others provide food for humans (Van Huis, 2003) or are a source of wild silk production (Kakati & Chutia, 2009), and some also are important for public health because of venomous or urticating setae on larvae and/or adults (Wirtz, 1984). The taxonomy and higher phylogeny of Saturniidae are reasonably well known (Regier et al., 2008), and they have been the subject of detailed taxonomical and ecological studies (Bernays & Janzen, 1988; Blest, 1960a,b; Janzen, 1984a,b; Janzen et al., 2012; Lamarre et al., 2015). Adult saturniids usually are nonfeeding, short‐lived moths with lightweight fast‐flying males adapted to find heavy‐bodied females (Scoble, 1995). Saturniid caterpillars tend to be relatively polyphagous and to prefer old, tough, tannin‐rich leaves (Bernays & Janzen, 1988). In contrast to other moth families such as Crambidae, Erebidae, or Geometridae, local species pools of Saturniidae are rather restricted, usually with <100 species in the Neotropics (Janzen et al., 2012; Lamarre et al., 2015; Lamas, 1989). The relatively low local species richness of Saturniidae facilitates the compilation of exhaustive lists for comparative ecological studies and long‐term monitoring.

In recent years, climate fluctuations and anomalies have triggered changes in local community composition and dynamics in Panama and, particularly, on Barro Colorado Island (BCI). More than 20 years ago, Condit, Hubbell, and Foster (1996) demonstrated the strong impacts of severe drought on the tree communities of BCI. In particular, El Niño is responsible for variability in fruit production and famine among frugivorous animals (Wright, Carrasco, Calderón, & Paton, 1999). The increase in air temperature and rainfall on BCI during the last twenty years has enabled a long‐term increase in liana abundance and tree flower production (Anderson‐Teixeira et al., 2014). A 40‐year monitoring of a lizard species on BCI indicates an alarming decline in population size (Stapley, Garcia, & Andrews, 2015).

In 2009, the ForestGEO (Forest Global Earth Observatory) Arthropod Initiative started long‐term monitoring among multiple contrasting taxa, including saturniid moths, within and near the permanent botanical plot of the Center for Tropical Forest Science (CTFS) on BCI. The program aims to detect long‐term changes in the abundance and composition of focal arthropod assemblages, driven primarily by climatic fluctuations and anomalies, as opposed to short‐term stochastic changes (Anderson‐Teixeira et al., 2014; Basset et al., 2013, 2015). Our present contribution reviews the minimum information necessary to discuss local saturniid assemblages in the tropics and relatively long‐term changes in their population dynamics, hence developing a framework onto which other recent time‐series of tropical arthropods may be similarly characterized and studied. Our main working hypotheses include the following: because Saturniidae are rather polyphagous (Bernays & Janzen, 1988), we would expect a limited effect of fluctuations in plant resources associated with climatic anomalies on their population dynamics, but perhaps not of temperature and of rainfall. The latter is known to affect caterpillar mortality in Panama (Caldas, 1992). Hence, we contend that Saturniidae on BCI may be sensitive in the long‐term to local changes in rainfall rhythm and intensity and that the magnitude of these effects may be severe for species that are rather host specific (i.e., less polyphagous). In addition, we might expect small‐bodied moths to be more sensitive to climate fluctuations because of higher dispersal limitation than larger species. Finally, species exhibiting strong seasonality patterns might also be impacted by shifts in season magnitude and strength.

The specific objectives of this study were twofold. (1) To establish baseline data for assessing long‐term changes in saturniids on BCI, (a) providing a revised checklist of the saturniids of BCI, with most species being characterized by their DNA barcodes (Hebert, de Waard, & Landry, 2010; see methods) and including older Panamanian records (1958–2016); (b) building a local phylogenetic tree for the saturniid species occurring on BCI; and (c) constructing a reproducible functional classification among the most common species using functional and ecological attributes describing dispersal ability, seasonality, and host specialization. Further, we will estimate total species richness on the island, discuss the composition of the saturniid assemblage, and test for independence between our functional classification and saturniid phylogeny. (2) To use the recent monitoring data to discuss possible changes in population trends for saturniids during the last eight years on BCI (2009–2016), by first, (a) testing for changes in annual species composition; (b) testing for significant population changes since 2009, either for species, higher taxa, or functional groups; (c) for the same datasets, testing for significant predictors of saturniid abundances during the study period; and finally, (d) evaluating which of the datasets (species, higher taxa, and functional groups) appear best suited to detect changes in population dynamics and interpret them. In sum, our study proposes a comprehensive framework for the study of long‐term monitoring of arthropods in tropical rainforests.

## 2 MATERIALS AND METHODS

### 2.1 Study site and compilation of saturniid data

Barro Colorado Island (9.15°N, 79.85°W; 120–160 m asl) in Panama receives an average annual rainfall of 2,662 mm, with an annual average daily maximum and minimum air temperatures of 31.0°C and 23.6°C, respectively (http://biogeodb.stri.si.edu/physical_monitoring/research/barrocolorado). An air temperature increase of 2–5°C is projected for Panama by 2080 (Stocker, Qin, & Platner, 2014). Since 1981, BCI has experienced an increase of 0.36°C in mean annual temperature and 17.9% in mean annual precipitation (Anderson‐Teixeira et al., 2014). During the study period, annual mean maximum and minimum temperatures rose slightly, especially since 2011 (Fig. S1). For example, the annual maximum temperature of 2016 was 0.6°C higher than that of the base year, 2009. However, annual rainfall did not appear to increase during the study period (Fig.S1). A detailed description of the setting and climatic data for the 50 ha CTFS plot, which is located in the center of the island, may be found in Windsor (1990), Condit (1998), and Anderson‐Teixeira et al. (2014). Around 1910, the Chagres River was dammed to fill the Panama Canal. Cerro Barro Colorado, cut off from the mainland by the rising water, became a 1,542 ha island (M. Solano pers. comm.). The island is entirely covered by lowland tropical forest with some anthropogenic clearings, locations of Smithsonian facilities.

We used eight datasets to compile records of Saturniidae collected or observed on BCI (Table 1) and verified saturniid taxonomy with Rougerie (2008). The datasets represent a mix of published lists, online databases, and rearing and monitoring projects (Table 1). The total 1,601 records are distributed from 1958 to 2016, but we will also refer later to the survey by Dyar (1914), predating the formation of BCI. The ForestGEO Arthropod Initiative used a light trap protocol to attract Saturniidae with traps set at 10 locations and run for four surveys each year (640 night samples; methods in Lucas, Forero, & Basset, 2016; and Appendix S1). These surveys were performed with a weighted frequency of dry/wet periods, so that annual moth population indices reflected well the whole year: dry period, ca January to April, one survey in March; wet period, ca May to December, three surveys in May, September, and November. Further and as far as possible, the May survey was performed 3 weeks after the official start of the wet season as reported by The Panama Canal Authority (data courtesy of The Panama Canal Authority; the average date of wet season onset is 4 May [1954–2016]).

**Table 1:** Datasets used to compile a list of species of Saturniidae collected or observed on BCI, 1958‐2016, with the number of records (individuals) and species for each dataset. Data from Dyar (1914) predate the formation of BCI and are detailed for comparison only

Dataset	Description	Reference	Records	Species
Dyar^a^	Published list, 1911‐1923 records	Dyar, 1914;	54	27
USNM^a^	Online database, National Museum of Natural History, Washington	USNM, 2017	6	6
Blest	Published list, 1958 records	Blest, 1960b;	20	20
STRI^a^	Online database, STRI synoptic dry collection, 1970‐2013 records	STRI, 2017	72	28
Aiello^a^	A. Aiello's rearing records on BCI, 1977‐2007	Aiello, 2017;	45	15
Coley^b^	P.D. Coley & T.A. Kursar rearing records on BCI, 1996‐2005	Dyer et al., 2007;	33	8
Symb^a^	Online database, Neotropical Arthropods Portal	Symbiota, 2017	73	11
Lemaire	Taxonomic works, no record years available	Lemaire, 1978, 1980, 1988, 2002;	10	10
ForestGEO	ForestGEO Arthropod Initiative, monitoring 2008‐2016	Basset et al., 2013;	1,288	46
Total			1,601	72^c^

a Including also Panamanian records other than BCI.

b Several records with year obscure, coded as 2000, the midpoint of 1996‐2005.

c Total number of species recorded for BCI.

### 2.2 Species identification

The ForestGEO data identifications relied on both morphological and molecular data. Of 1,274 specimens collected in light traps, we pinned and retained 646 specimens (i.e., 51%). The remainder was discarded after identification. First, specimens were identified morphologically using Janzen and Hallwachs (2016) and using more specific publications and expert opinion when needed (RR, TD). Second, DNA Cytochrome c oxidase subunit I (COI, “DNA barcode”) sequences were processed from the ForestGEO pinned specimens at the Biodiversity Institute of Ontario, University of Guelph, using methods described in Wilson (2012). In total, we obtained 167 sequences. Molecular data were used to confirm identifications based on morphology and to examine the possibility that morphological uniformity might conceal cryptic species. All species delineated by molecular data are unambiguously referred to by their Barcode Index Number (BIN), which can be used as a proxy taxonomic unit (Ratnasingham & Hebert, 2013). Morphospecies not yet formally described but with distinct BINs are termed “cryptic” species, even if in some cases they can be distinguished morphologically from other species. See Appendix S1 for further details.

### 2.3 Species pool, faunal composition, and phylogenetic relationships

We estimated the species richness of saturniids on BCI with two methods. First, we estimated the total number of species likely to be present in the understorey of BCI forests by randomizing 100 times the cumulative number of species collected/observed during 32 surveys performed during 2009–2016 by ForestGEO (i.e., 640 trap‐nights) and calculating the Incidence Coverage‐based Estimator (ICE) with EstimateS 8.20 (Colwell, 2009). Although light traps were located in the undestorey, the accumulation of saturniid species during the study period (Results, Fig.S2a) suggests that the canopy stratum was also well sampled, presumably because saturniid moths are strong fliers that can be attracted to the lower strata. Second, we fitted a nonlinear regression with the software CurveExpert (Hyams, 2011) to the cumulative number of individuals sequenced against the cumulative number of cryptic species discovered, in order to appreciate how many cryptic species remain to be discovered on BCI. We choose the best model according to the highest *R* ^2^ and the lowest corrected Akaike's Information Criterion (AIC). We also used nonmetric multidimensional scaling (NDMS) to compare the faunal composition of the key datasets and years of our compilation (Table 1): Dyar, Blest, Aiello, and ForestGEO, years 2009 to 2016 (matrix of 80 species × 9 datasets; Bray–Curtis distance). The similarity of assemblage composition among collecting years was estimated with the Morisita–Horn index, comparing abundance data, routinely used in entomology (Magurran & McGill, 2011), and calculated with the R‐language function vegdist of the vegan library (Oksanen et al., 2011). We attempted to explain the signification of the NDMS axes for ForestGEO data with correlation between the scores of Years and the abundance of all species collected per year or the abundance of certain subfamilies collected per year. We obtained DNA barcodes from 58 of the 72 saturniid species recorded in the study for inclusion in our phylogenetic analysis. Together, these 58 species represent 95% of individuals observed on BCI. We used a tree‐grafting approach to estimate a community‐level phylogeny. Details about the software used for these various analyses are indicated in Appendix S1.

### 2.4 Saturniid functional attributes and classification

We first used a combination of morphological traits to estimate the ability of a species to disperse (Sekar, 2012) and to shift to different habitats in a changing environment (Diamond et al., 2011; Slade et al., 2013). We measured forewing length (FW) and thorax width (TW) for each species and calculated the ratio of TW to FW as an index of flight strength (Wing load). We then build an index of seasonality representing the variance around the peak of maximum abundance of each species per year (hereafter Var_Peak), over the 8 years of monitoring (Lamarre, Mendoza, Fine, & Baraloto, 2014). A low variance around the peak of maximum abundance denotes a narrow peak of abundance and a strong annual seasonality. Note that quarterly surveys within years allow only a minimal resolution in the estimate of seasonality, but are enough to identify dry or wet season specialists. We also estimated an index of relative host specialization for each taxon (DBIF) as the proportion of host plant families used by the species relative to the total number of host plant families recorded in the database. A small diet index indicates high specialization for saturniid species feeding on fewer host plant families. Calculations of these variables are detailed in Appendix S1.

We were able to quantify functional attributes for 41 species and create a matrix of species × functional traits that included five variables: FW, TW, Wing load, Var_Peak, and DBIF (Appendix S2). We used this matrix to compute a principal component analysis (PCA). The different components (axes) of the PCA were used as combinations of traits to define a Euclidean functional space with reduced uncorrelated dimensions, where each species was represented according to its PCA scores. This approach is recognized as an efficient way to produce high‐quality functional spaces (Maire, Grenouillet, Brosse, & Villéger, 2015). We then computed a functional tree using hierarchical daisy clustering methods based on functional clusters defined by the species scores on all axes. Further details on these analyses are indicated in Appendix S1. Finally, we tested the hypothesis that this trait‐based approach represented a functional classification that was independent of moth phylogeny. We mapped each of our clustered functional groups onto the community phylogeny and calculated the mean phylogenetic distance (MPD) between all members of the same cluster. These mean values were compared to randomized distributions as generated by 999 tip label permutations; a two‐tailed test was used to assess the probability that mean values fell outside of either tail of the randomized distribution. Further details and software used for these analyses may be consulted in Appendix S1.

### 2.5 Moth population dynamics

To analyze recent population trends, we fitted to each species sufficiently well sampled (i.e., probability to have collected at least one individual per survey each of the 8 study years, total of individuals collected >32, *n* = 14 species) three different time‐series models (log‐linear Poisson regression models) with the software TRIM (Pannekoek & van Strien, 2005), using a matrix of species abundance at each location (*n* = 10) and year (*n* = 8). The models included the following: (1) no time‐effects: counts vary only across sites and not across time points; (2) linear trend: model with a site‐effect and a linear (on the log scale) effect of time; and (3) model with separate parameters for each time point. For models (2) and (3), TRIM calculates a multiplicative slope, which corresponds to the yearly change and is converted into one of the following categories: strong or moderate increase, stable, uncertain, moderate, and steep decline (Pannekoek & van Strien, 2005; details in Appendix S1). Goodness of fit of the models was assessed with chi‐square tests, and the model with the lowest Akaike information criterion score (AIC) was selected. We ran TRIM models for the most common species, for higher taxa together (saturniid family and subfamilies), and for functional groups as delineated by our cluster analysis.

Further, we explored the attributes of our time‐series with tools in the package Systat 13.1 (Systat Software Inc., Chicago, Illinois, USA, 2009). First, we used autocorrelation function analysis and partial autocorrelation function analysis plots (Box & Jenkins, 1976) to examine whether time‐series were autocorrelated or not. Second, because most of our time‐series were sinusoid shaped with a seasonal component, we used a Fourier transformation on raw data and modeled them with autoregressive integrated moving average models (ARIMA; Hamilton, 1994). We built forecast models for eight new surveys (predicted for the next 2 years) with an ARIMA with seasonal periodicity of four surveys for the same datasets used in TRIM analyses. Because most time‐series were not autocorrelated, we then fitted stepwise multiple regressions to the log(x + 1) transformed abundances of the same datasets, using a variety of climatic independent variables, including sea surface temperatures that characterize El Niño and La Niña events. These variables are detailed in Table S1 and fall into three categories as follows: (1) variables related to the conditions of the day of the survey; (2) variables related to the month of the survey; and (3) variables with a time lag of 15 or 30 days preceding the survey (see details in Lucas et al., 2016). Because insect development is temperature dependent, the accumulation of degree‐days is used routinely in entomology and is relevant in the context of predicting changes in herbivore populations (e.g., Ives, 1973 for pests). Hence, we considered the accumulation of degree‐days as explanatory variables in the (3) category and calculated them as indicated in Table S1. Another important variable in category (3) was the El Niño/Southern Oscillation (ENSO) index, obtained from the National Oceanographic and Atmospheric Administration (2016). Although we removed variables with high multicollinearity from the final models (i.e., when tolerance values >0.001), models should be viewed as explanatory analyses. Eventually, we performed a canonical correspondence analysis (CCA) to evaluate the influence of climatic variables (Table S1) on the composition of saturniid species within each survey (details in Appendix S1).

To sum up these various analyses, the TRIM models aimed to test whether, (1) changes in abundance were similar in all spatially replicated locations (and therefore more likely to reflect real population changes as opposed to changes related to the conditions of the surveys); and (2) significant increase or decrease in abundance existed, compared to the base year, 2009. On the other hand, multiple regressions and the CCA aimed at testing the relative influence of climatic variables on changes in moth abundance and species composition, respectively. Finally, the ARIMA models attempted to forecast two future years of data according to the structure of our existing data.

## 3 RESULTS

### 3.1 Taxonomic and historical knowledge

Altogether, we recorded 72 species of saturniids on BCI for the period 1958 to 2016 (Appendix S2), representing ca. 60% of the currently estimated diversity of saturniid species in Panama. Most species were named (88.9%) and had valid BINs (80.6%), including 6.9% of cryptic species, 56.9% of species with sequences originating from BCI, and 43.1% of species with sequences originating from other tropical sites. Dyar (1914) collected 27 species in the Canal Zone, while the ForestGEO monitoring recorded 41 species with light traps on BCI (Fig.S2a). The accumulation of singletons for the ForestGEO light trap data suggests that at least 44.1 ± 0.01 species (ICE ± SD) may be collected by light traps on BCI (Fig.S2a). However, four cryptic species were discovered after sequencing 167 individuals. The best‐fit model between the cumulative number of individuals sequenced and the cumulative number of cryptic species discovered was a reciprocal logarithm model, suggesting that many more cryptic species remain to be discovered on BCI, with accrued numbers of specimens being sequenced (Fig.S2b).

**Figure 2:**
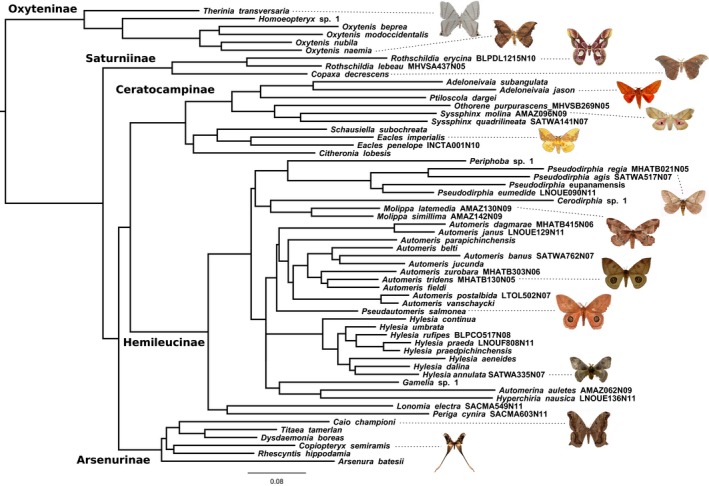
The best‐scoring maximum likelihood tree depicting the phylogenetic relationships among 58 species of saturniid moths occurring on BCI. Scale bar in substitutions per site. Sequence codes are indicated for species not barcoded by ForestGEO

We were able to obtain at least five DNA sequences for most saturniid species, which were well delineated by DNA barcodes (average normalized divergence within species 0.09%, Table S2). In the ForestGEO dataset, saturniid genera were delineated by at least 9.5% of divergence (Table S2). We recovered phylogenetic hypotheses in general agreement with published studies (Regier et al., 2008), despite the fact that over 50% of taxa in the final analysis were sequenced only for COI. However, as expected, COI was not totally effective at resolving deeper relationships. *Eacles penelope*,* Gamelia* sp. 1, *Periphoba* sp. 1, and *Periga cynira* had to be constrained to occur in their respective subfamilies, and *Automeris* had also to be constrained to be monophyletic. A phylogenetic tree for the most common species on BCI is presented in Figure 2, and supporting data are included in Appendix S3.

**Figure 3:**
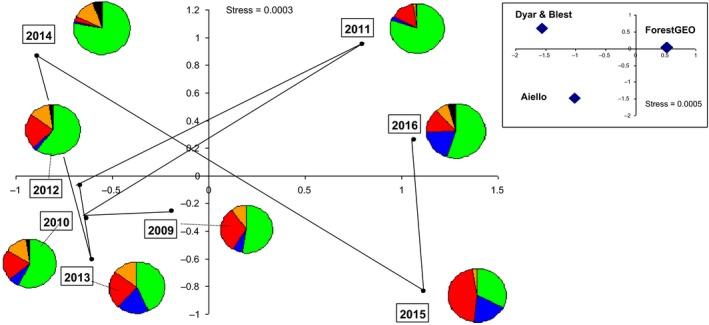
Plot of the scores for ForestGEO sampling years on axes 1 and 2 of the NMDS. Years are linked chronologically by a solid line. Pie charts indicate for each year the proportion of abundance accounted by (in clockwise order) Hemileucinae (green), Ceratocampinae (blue), Arsenurinae (red), Oxyteninae (orange), and Saturniinae (black). Inset: plot of the scores of the Dyar, Blest, Aiello, and ForestGEO datasets (2009‐2016) in axes 1 and 2 of the NMDS. Dyar and Blest have similar scores, and likewise all ForestGEO years are grouped together

The NDMS analysis clearly separated recent monitoring years (2009‐2016) from older datasets (Dyar and Blest) and rearing records (Aiello; inset of Figure 3). The NDMS also clearly separated ForestGEO monitoring years, with the most dissimilar pairs of years being 2010‐2011 and 2014‐2015 (main Figure 3). Axis 1 of the ordination was correlated with the total number of individuals collected each year (*r* = .846, *p* = .008), with Years 2011, 2015, and 2016 yielding high numbers of catches. Axis 2 of the ordination was correlated with the coefficient of variation of abundance in each subfamily for each year (*r* = .905, *p *= .002), with, for example, a high proportion of Hemileucinae collected in Years 2014 and 2011, relative to the overall total of specimens trapped per year (main Figure 3). We also note that the lowest overall faunal similarity was between a pre‐El Niño year (2014) and an El Niño year (2015, Table S3).

### 3.2 Functional classification

**Figure 4:**
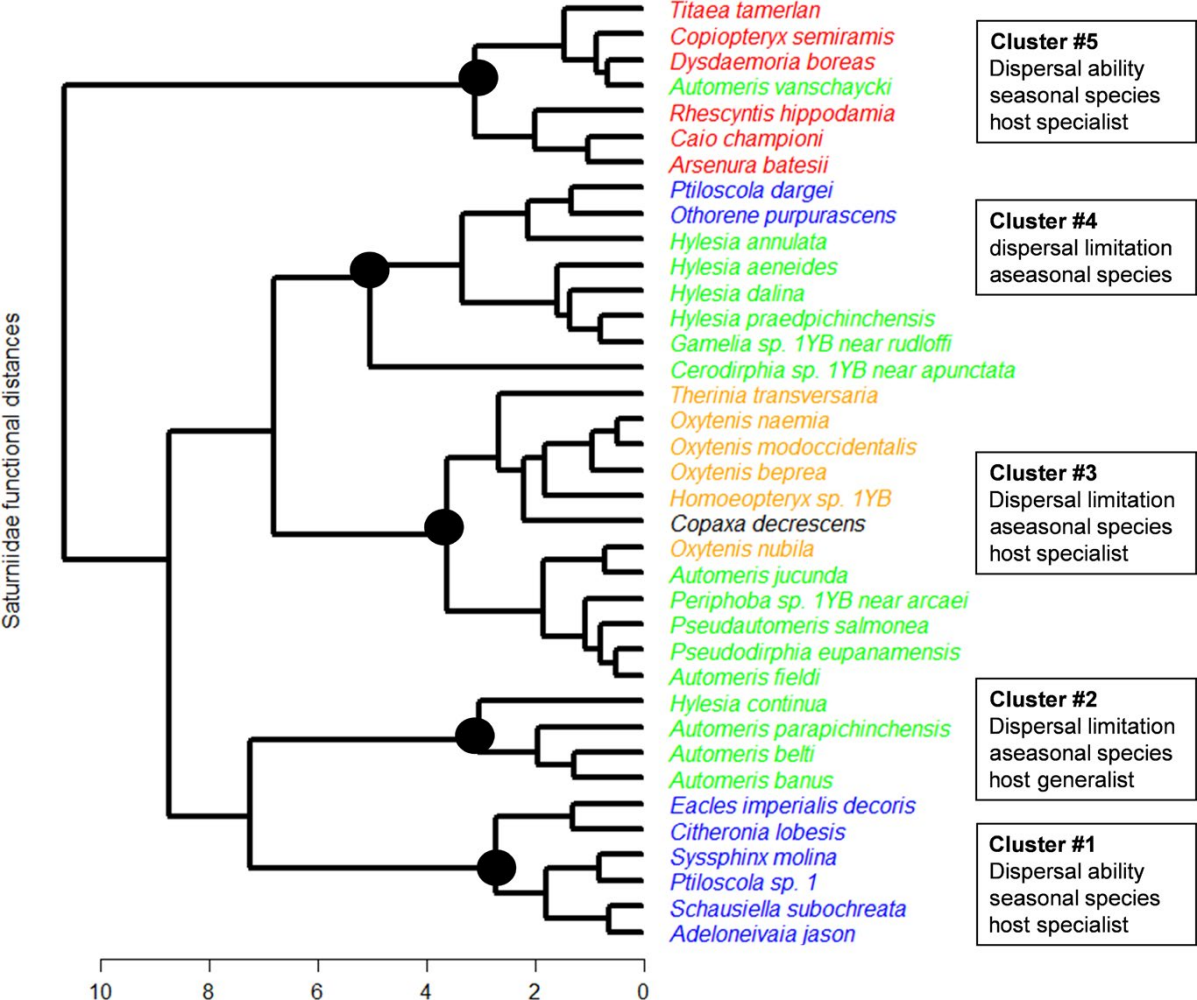
Cluster of functional groups of the BCI Saturniidae community. Saturniidae species are colored (as in Figure 3) to visualize phylogenetic conservatism in functional groups. Boxes describe the main functional characteristics of each group (Table S4). Our interpretation for each group is best illustrated using the Euclidian functional space presented in Fig.S2

Saturniid species that share life‐history traits related to dispersal, annual seasonality, and host plant specialization were clustered into five distinct functional groups (Figure 4). Saturniid species in Cluster #1 and #5 are best‐represented by large body‐size species (i.e., high dispersal ability) showing a high annual seasonality (i.e., species that showed convergent monthly peaks of abundance over the 8‐year study period) and to a lesser extent high host specialization (specialists; Figure 4, Table S4). Species grouped in Cluster #5 were among the most specialized saturniids on BCI (see Fig.S3, axis3). Cluster #2 is composed by small‐ to medium‐size Hemileucinae (i.e., dispersal limitation) presenting a low annual seasonality and the most host generalist species. We recorded the use of 45 distinct host plant families by *Hylesia continua* and *Automeris banus* (Cluster #2, Figure 4). A group of species in Hemileucinae and Oxyteninae form Cluster #3, including host specialist species with potentially high dispersal limitation due to their small body‐size. Species of Oxyteninae were the most host specialized saturniids in our collection with no more than two host plant families recorded. Finally, species grouped in Cluster #4 are relatively aseasonal and have a very low degree of host specificity (Table S4). Overall, our functional classification best interpreted by the Euclidian functional space (Fig.S3) distinguished in Axis 1 the species with large body‐size (dispersal ability) and seasonal pattern versus smaller body size (dispersal limitation) showing aseasonal patterns (Cluster #1 and #5 vs. Cluster 2#3#4). In addition, axes 2 and 3 separated species showing higher host specificity (Clusters #3, #5) from species that were more host generalists (Clusters #1, #2). Two of the five functional groups (Clusters #2 and #5) represented clustered subsets of the saturniid community phylogeny, while one was marginally significant (Cluster #1; Table S4, Fig. S4).

### 3.3 Moth responses to climatic events

**Figure 5:**
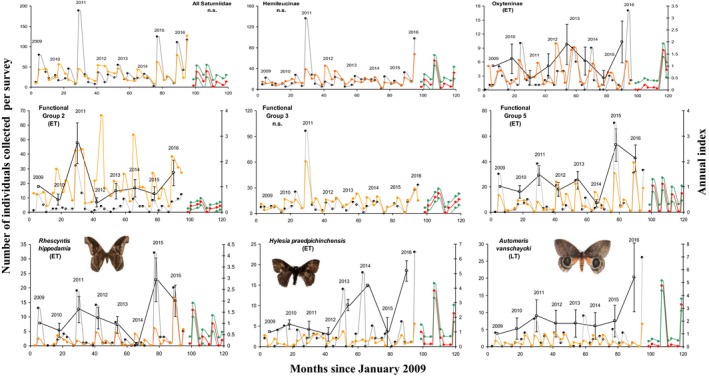
Population changes on BCI during 2009‐2016 for top row: all Saturniidae and selected subfamilies; second row: selected functional groups; and third row: selected species. Gray lines: raw data; black lines: fitted TRIM models [±SD] (Table 2); dashed orange lines: fitted regressions (Table S5); dashed red lines: forecast of ARIMA models for 2017‐2018; and dashed green lines: 95% confidence limits of ARIMA models. Other taxa and groups are similarly plotted in Fig.S4

No significant TRIM model could be fitted to the time‐series of all specimens of Saturniidae (Table 2, Figure 5), hardly surprising as most species had different population trends during 2009‐2016. Of the 14 species that could be tested, three could not be fitted to any of the TRIM models (Table 2, Figure 5, Fig.S5). Two species (14% of species tested) showed a significant increase over time, namely *Hylesia praedpichinchensis* and *Automeris vanschaycki*. The rest of the species (64%) showed significant effects for each time point but the direction of the change was uncertain. Six species showed a clear peak in abundance in 2011, four species in 2016, three species in 2015, and this was reflected when all specimens of Saturniidae were considered together. Whereas TRIM models could be fitted to only one (Oxyteninae) of five saturniid subfamilies, they could be fitted to two of five functional groups. For functional Cluster #2, the direction of change was uncertain, whereas functional Cluster #5 showed a significant increase in time (Table 2, Figure 5, Fig. S4). The multiplicative slope estimate calculated by TRIM for the 11 species with significant models could be explained by a weak but significant multiple regression explaining 42.3% of the variance: slope = 0.666 + 0.006 × forewing length + 0.162 × Var_Peak (*F*_2,8 _= 4.66, *p* = .046), in which forewing length had a large and positive standard coefficient.

**Table 2:** Summary of models fitted by TRIM (see text) to the time‐series (2009‐2016) of saturniids for higher taxa (too few Saturniinae were collected for analyses), functional groups, and the most common species (listed in decreasing order of abundance, see Appendix S2)

Taxa/Cluster/Species	Best model	Chi‐square *p*	AIC	Overall mult.	*SE*	Interpretation
				slope	slope	
All Saturniidae	None of models significant	‐	‐	‐	‐	‐
Arsenurinae	None of models significant	‐	‐	‐	‐	‐
Ceratocampinae	None of models significant	‐	‐	‐	‐	‐
Hemileucinae	None of models significant	‐	‐	‐	‐	‐
Oxyteninae	Effect for each time point	0.074	−35.14	1.041	0.058	Uncertain
Functional Cluster # 1	None of models significant	‐	‐	‐	‐	‐
Functional Cluster #2	*Effect for each time point*	0.216	−46.79	1.03	0.0415	Uncertain
Functional Cluster #3	None of models significant	‐	‐	‐	‐	‐
Functional Cluster #4	None of models significant	‐	‐	‐	‐	‐
Functional Cluster #5	Effect for each time point	0.062	−31.59	1.091	0.032	Moderate increase (p < 0.01)**
*Rhescyntis hippodamia*	Effect for each time point	0.339	−48.07	1.301	0.055	Uncertain
*Hylesia praedpichinchensis*	Effect for each time point	0.171	−46.52	1.189	0.066	Strong increase (p < 0.05) *
*Automeris parapichinchensis*	Effect for each time point	0.328	−50.79	1.017	0.052	Uncertain
*Automeris vanschaycki*	Linear trend	0.404	−58.91	1.159	0.059	Moderate increase (p < 0.01) **
*Periphoba* sp. 1YB	Effect for each time point	0.061	−43.02	0.962	0.059	Uncertain
*Citheronia lobesis*	None of models significant	‐	‐	‐	‐	‐
*Automeris fieldi*	Effect for each time point	0.328	−57.62	1.19	0.176	Uncertain
*Pseudodirphia eupanamensis*	Effect for each time point	0.463	−63.75	1.022	0.082	Uncertain
*Pseudautomeris salmonea*	None of models significant	‐	‐	‐	‐	‐
*Oxytenis naemia*	Effect for each time point	0.529	−65.13	0.896	0.127	Uncertain
*Automeris belti*	Effect for each time point	0.122	−53.66	0.999	0.085	Uncertain
*Caio championi*	Effect for each time point	0.946	−70.29	1.085	0.109	Uncertain
*Titaea tamerlan*	None of models significant	‐	‐	‐	‐	‐
*Oxytenis beprea*	Effect for each time point	0.201	−74.75	0.998	0.111	Uncertain

ARIMA models predicted seasonal peaks for 2017 and 2018 (as expected), but never of the magnitude of years 2011, 2015, or 2016 (Figure 5, Fig.S5). More than half of models (54%) predicted seasonal peaks larger than those of the 2009 base year. Only two of the 24 time‐series tested presented some degree of autocorrelation. Although stepwise regressions accounted for 20%–75% (average 45%) of the variance in log abundance of saturniids per survey, they were not accurate enough to predict the peaks in abundance of year 2011, 2015, and 2016 for many taxa (Table S5, Figure 5, Fig.S5). Overall, climatic variables accounting for time lags or reflecting conditions during the month of the survey were better predictors of saturniid abundance than variables relevant to the day of the survey (Table S5). One of the most consistent predictors of saturniid abundance was the accumulation of degree‐days during the 30 days preceding the survey (Table S5, best predictor in 54% of regressions, positive coefficient in most cases). Similarly, the average minimal air temperature during the 30 days preceding the survey was also a good predictor of saturniid abundance (negative coefficient in all cases). The sum of rainfall during the month of the survey was also a relatively good predictor, but its influence on saturniid abundance could be either positive or negative, depending on the cases. We also note that soil humidity and solar radiation for the month of the survey contributed positively and negatively to saturniid abundance, respectively, and were possible surrogates for vegetation growth. In contrast, the influence of the monthly ENSO index was not obvious.

Six climatic variables (Table S1) explained 39.9% of variance in faunal composition among surveys in the CCA (test for significance of all axes, *F* = 2.8, *p* = .002; Fig.S6). The sum of degree‐days during the 30 preceding days of the survey best explained the formation of the first Axis of the CCA. Soil humidity explained best the formation of the second axis. The average minimal temperature during the 15 days preceding the survey and the average moonlight during the survey was best correlated with axes 3 and 4, respectively. Interestingly, climatic variables with a time lag prior to the survey were more likely to explain the variance in faunistic composition than variables related to the climatic conditions of the survey, but we acknowledge the possible influence of moonlight in this context.

## 4 DISCUSSION

### 4.1 Monitoring program

Light traps represent a relatively easy and convenient way to collect Saturniidae in tropical rainforests (Lamas, 1989). However, light trap catches are heavily biased toward males (83% of individuals in the ForestGEO data) that are actively seeking females. This may or may not impede sound interpretation of the data, depending on the objectives of the study. For the purpose of long‐term monitoring, this appears not to be problematic. More questionable for saturniid surveys is the low efficiency of automatic bucket traps to collect the largest species, such as *Rothschildia* spp., which were reared on BCI (Aiello records) but rarely collected in ForestGEO light traps. One remedy would be to increase the diameter of the lower funnel hole (6 cm in ForestGEO traps) and the capacity of the collecting bucket.

It is also well known that light traps measure the density in flight activity for moths, dependent on air temperature, humidity, moonlight, etc., which may not necessarily be correlated with actual population abundance (Muirhead‐Thomson, 1991). In our study, we note that climatic variables explaining the abundance of saturniids within surveys rarely included variables related to the dates of light trapping but, rather, variables with time lags of 30 days. This suggests that our data reflect real changes in population dynamics rather than mere changes in moth activity, an idea clearly supported by the high number of species tested whose time‐series could be fitted with TRIM models accounting for the effects of time at each location. This effect would have been less obvious if species abundances were entirely dependent on local trap conditions (e.g., effect of background trap illumination in the forest) and in turn more likely to vary differently among sampling locations. Further, the four surveys performed per year with a weighted frequency of dry/wet periods represented a good compromise between sampling effort and precision in measuring fluctuations in moth abundances and allowed us to derive representative moth population indices for the whole year, accounting for seasonal effects (Basset et al., 2013). However, our protocol cannot address the interactions between changes in land use and climate change (Agosta, Hulshof, & Staats, 2017).

### 4.2 Size and composition of the saturniid assemblage

The accumulation curve for the ForestGEO light trap data suggests that the saturniid fauna has been relatively well sampled as at least 44 species were present at the fixed locations where the traps were running. With respect to estimating the local species pool on BCI, this finding is misleading for at least two reasons. First, our collection refers only to the shady understorey of the forest, where attraction of saturniids to light is probably <50 m (Beck & Linsenmaier, 2006). Moving the ForestGEO light traps to other locations on BCI may increase the number of species collected, as suggested by the list of the 72 species recorded altogether on the island. Second, accumulation of cryptic species is strongly dependent on the number of specimens sequenced, and we were able to sequence only a small proportion of the material collected by ForestGEO light traps (13%). The present saturniid list for BCI currently includes only four cryptic species (6% of the total), including one species that was common enough to be well monitored during the period 2009–2016. Janzen et al. (2012), after sequencing a massive 48,000 + saturniid specimens from Guanacaste (Costa Rica), showed that the proportion of cryptic species was 40%. Therefore, a species pool of 60 + breeding species on BCI looks plausible.

### 4.3 Saturniid population dynamics

There have been very few long‐term monitoring studies of Lepidoptera in tropical rainforests, with exceptions such as Leidner et al. (2010), Grøtan et al. (2012), and Valtonen et al. (2013). In the tropics, insect species commonly produce eight generations every year (Nair, 2007). Polar, Cock, Frederickson, Hosein, and Krauss (2010) mention four generations per year for *Hylesia* spp. Data from Janzen (1984b) suggest between three and six generations per year. This figure suggests that our short time‐series of 8 years for BCI saturniids nevertheless represent >30 saturniid generations for most species. Both the data on faunal composition (Figure 3) and the time‐series (Figure 5, Fig. S5) suggest that year 2011 was rather different from other years, with distinct peaks in populations for six of the 14 saturniid species studied. Other peaks of saturniid abundances occurred mainly in 2015 and 2016. Although the relationship between monthly ENSO and saturniid abundance is not straightforward (as judged by our multiple regressions, Table S5), we note that years 2011, 2015, and 2016 are qualified as weak‐to‐moderate La Niña event, very strong El Niño event, and very strong El Niño event, respectively (National Oceanographic and Atmospheric Administration 2016). The number of saturniid species peaking each year appears to be related to the magnitude of these events, which also is reflected in changes in faunal composition, as illustrated by NDMS plots (Figure 3), with Axis 1 clearly separating these three years from other sampling years. The lowest overall faunal similarity was also between a pre‐El Niño year (2014) and an El Niño year (2015, Table S2). Our results suggest that saturniid abundances and composition are affected by El Niño and La Niña events, but that longer time‐series including more of these events are certainly needed to build a comprehensive understanding of insect responses to such climate anomalies.

The time‐series for most saturniid species tested showed significant effects for each time point. However, the often‐abrupt yearly changes in abundance for these species precluded establishing the direction of change. Yet many species were more abundant in 2016 as compared to the 2009 base year, and ARIMA forecasts for the next two years suggest that this pattern will hold for many species. The populations of only two species, *Automeris vanschaycki* and *Hylesia praedpichinchensis*, showed significant increases over time during the study period. Neotropical *Hylesia* are known to produce population peaks and even outbreaks (Janzen, 1984a; Polar et al., 2010), and the present observation may be related to this ability. Our simple models based on stepwise multiple regressions with climatic variables were able to some extent to predict saturniid abundance, but they could be improved using synthetic climatic variables and other variables accounting for biotic factors. Unfortunately, to date, there is no index of vegetation phenology that can be suitable at the community level for BCI (S.J. Wright pers. comm.). Our regressions suggest that in many cases, the accumulation of degree‐days during the 30 days preceding the survey was a relevant and positive predictor of saturniid abundance. The influence of rainfall in this respect was more equivocal. Hence, we expect that the current increase in air temperature observed on BCI (Anderson‐Teixeira et al., 2014) will lead to an overall increase in many saturniid species in the long‐term, all other things being equal.

### 4.4 Merit of considering functional groups in monitoring programs

As compared with other insect groups, Saturniidae overall appear rather homogeneous as far as life history is concerned, because they are almost all relatively polyphagous as larvae and short‐lived as adults. However, our study showed that they display contrasting functional attributes and that functional groups may exhibit different time‐series, possibly related to climate changes or extreme events. For example, functional Cluster # 5 included large species with good dispersal ability, relative host specificity, and seasonality, and those species collectively showed a significant and moderate increase over time, as compared to the 2009 base year. This pattern is also demonstrated by the positive relationship between the multiplicative slope calculated by TRIM models, related to an increase in population abundance, and forewing length. Hence, we conjecture that the saturniid population increase expected in future years, as discussed in the previous section, would be reflected primarily by species included in functional Cluster # 5, a result suggesting that rising air temperature may more directly affect larger species. It would also support our prediction that the effect of climate fluctuation on species restricted to a smaller set of host plants (i.e., specialist) might be stronger. However, we found little support for direct effects of rainfall on small dispersal‐limited saturniid species such as functional Clusters #2 and #3.

Species from cluster #5 likely have many common traits due to shared ancestry (with the exception of *Automeris vanschaycki*), suggesting also that the subfamily Arsenurinae as a whole may be a useful source of indicator species. They cannot be considered as fully phylogenetically independent in their climactic response, as the traits explaining this response maybe correlated between species. It is possible that a trait correlated with body size is the main driver of their response to climate. However, our analysis also identified that the slope of increase in abundance in TRIM models was also correlated primarily with forewing length. We suggest that we have isolated one of the key traits for predicting population increases: body size, with the added advantage that taxonomy may be used to select further indicator species, but acknowledge that wider sampling may help test the generality of this conclusion.

Adult saturniids do not feed, have short life spans (often 4 to 14 days), and, particularly for the heavy‐bodied species, are unable to fly until they first have raised the temperature of their thoracic muscles to 35°C by a period of “shivering” (Blest, 1960a). For saturniids, rising air temperatures may mean allocating less time to shivering and more time available for mating partners and searching for suitable oviposition sites and may represent a significant advantage for species with particularly short adult life spans (Janzen, 1984b). We speculate that it may represent the basis for explaining the positive effect of rising air temperature on certain saturniid species, as observed for the time‐series of species included in functional Cluster # 5.

TRIM models were significant for 79% of species tested, 40% of functional groups, and 20% of higher taxonomic groups (subfamilies). The average percentages of variance explained in multiple regressions were also 43%, 54%, and 49% among species, functional groups, and subfamilies, respectively. Unquestionably, time‐series should be analyzed at the species level for an optimal interpretation of observed patterns. However, we imperatively need strategies to summarize these patterns conveniently when confronted with diverse groups such as insects. Our results suggest that the use of functional groups over taxonomic categories complement and improve our ability to interpret results, even for groups as ecologically homogeneous as the Saturniidae. Further, species traits that we compiled for each saturniid species are rather preliminary, as compared to those used for tropical ants (Parr et al., 2017). Improved species traits may result in improved resolution of functional groups. Our basic analysis of phylogenetic clustering revealed that the functional groups that were most suitable for time‐series analysis were also clustered across the community phylogeny. It is likely that groups, that share a close common ancestry, present uniform responses to environmental changes driven by physiological conservatism.

### 4.5 The use of saturniids for long‐term monitoring in tropical rainforests

Most of the rather large saturniid moths are charismatic and may be appreciated by the public; their taxonomy is relatively well advanced, they are easily collected by automatic bucket light traps, local species pools are probably often <100 species, and a few common species are amenable to statistical analysis of long‐term population trends, as shown in this study. The largest species cannot be collected easily with the trap model that we used, but this is not necessarily a strong limitation. The biology of saturniids also is interesting with regard to monitoring, as discussed previously. Overall, these various attributes appear rather positive to recommend saturniids as model taxa for studying the long‐term effects of climate change on tropical insects. However, the relative sensitivity of saturniid species to climate change in the long‐term is unknown and may be appreciated only when comparing saturniid time‐series to those of contrasting tropical insects popular in the conservation literature, such as butterflies, bees, and ants. We will discuss this issue elsewhere, as the ForestGEO monitoring data accumulated for BCI are ideally suited for that.

## DATA ACCESSIBILITY

All sequences are available online in the BOLD database (http://www.boldsystems.org/, project BCISA, public dataset https://doi.org/10.5883/ds-bcisat) and in GenBank (http://www.ncbi.nlm.nih.gov/genbank/, GenBank accession numbers: KP845288 ‐ KP845386). The occurrence of each species per year (1958‐2016) is detailed in Appendix S2, along with species traits. Data to reproduce the local phylogenetic tree are included in Appendix S3.

## CONFLICT OF INTEREST

The authors declare no conflict of interest.

## AUTHOR CONTRIBUTIONS

YB, GPAL, HB, and SEM conceived the ideas and designed methodology; TD, RR, FP, RB, YL, JAR, and AA collected the data; YB, GPAL, TR, and STS analyzed the data; YB and GPAL led the writing of the manuscript. All authors contributed critically to the drafts and gave final approval for publication.

## Supporting information

** **
