# Supplementary material for: The Saturniidae of Barro Colorado Island, Panama: A model taxon for studying the long‐term effects of climate change?
Source: Ecol Evol. 2017 Oct 22;7(23):9991–10004. doi: 10.1002/ece3.3515 (PMC5723595; doi:10.1002/ece3.3515)
Supplement: Supplementary file 1 [file ECE3-7-9991-s001.doc]

The Saturniidae of Barro Colorado Island, Panama: a model taxon for studying the long-term effects of climate change?

Supporting Information

**Appendix S1.** Supplementary methods.

#### Compilation of saturniid data

The ForestGEO arthropod Initiative used a light trap protocol to attract Saturniidae (see Lucas, Forero & Basset 2016). It consisted of 10W black light traps, fitted with acrylic transparent panes and an acrylic roof to protect catches from rain (Kitching, Li, & Stork 2001). Traps were set up in the forest understorey, at diameter breast height (i.e. 1.3m), in ten fixed locations on BCI. These locations were at least 300m distant from each other. One survey consisted of running a trap at each of the ten locations for two full but non-consecutive nights, yielding 20 night-samples. We performed four surveys in March (dry season), May (early wet season), and September and December (both wet seasons), for a total of 80 night-samples per year (total 640 night samples for 2009-2016). Trapping was performed only during new moons.

Species identification

So far 119 species of Saturniidae have been recorded from Panama (R. Rougerie, unpubl. data). Dyar (1914) collected 27 species of Saturniidae in the “Canal Zone,” during 1911-1913 and prior to the formation of Barro Colorado Island. Forbes (1942) in his “Lepidoptera of Barro Colorado Island” does not treat Saturniidae but indicates that Marston Bates had originally intended to study them. However, to the best of our knowledge, no such study was ever published. Blest (1960b) collected 20 species on BCI, whereas Annette Aiello reared about 15 species mostly from BCI (Aiello & Balcazar, 1997). In summary, there are very few published and updated lists of Saturniidae for Panama and for BCI, despite being one of the best-studied rainforest sites in the tropics (Leigh, 1999) and attracting hundreds of researchers each year.

As indicated earlier, we used Rougerie (2008) to update the taxonomy of older saturniid records from BCI. Following Braby, Eastwood & Murray (2012), we refrained from using subspecies, because they are inconsistently defined and frequently fail to reflect distinct evolutionary units according to population genetic structure. Voucher specimens were deposited into the collections of the ForestGEO Arthropod Initiative at the Smithsonian Tropical Research Institute and the reared collection of Aiello into the collections of the U.S. National Entomological Collection.

Note that the species that Aiello and Balcazer (1997) recorded from BCI as *Oxytenis modestia* was recognized as a new species, *Oxytenis modoccidentalis*, by Brechlin *et al*. (2014).

#### Species pool, faunal composition and phylogenetic relationships

We estimated the species richness of saturniids on BCI with two methods. First, we estimated the total number of species likely to be present in the understorey of BCI forests by randomizing 100 times the cumulative number of species collected/observed during 32 surveys performed during 2009-2016 by ForestGEO (i.e. 640 trap-nights) and calculating the Incidence Coverage-based Estimator (ICE) with EstimateS 8.20 (Colwell 2009). The ICE is one of the best nonparametric estimator of total species richness, and can produce stable estimates for subsets of the data (Colwell 2009). Second, we fitted a non linear regression with the software CurveExpert (Hyams, 2011) to the cumulative number of individuals sequenced against the cumulative number of cryptic species discovered, in order to appreciate how many cryptic species remain to be discovered on BCI. We choose the best model according to the highest R^2^ and the lowest corrected Akaike’s Information Criterion (AIC). We also used Non-metric multidimensional scaling (NDMS) to compare the faunal composition of the key datasets and years of our compilation (Table 1): Dyar, Blest, Aiello and ForestGEO, years 2009 to 2016 (matrix of 80 species x 9 datasets; Bray-Curtis distance). We used WinKyst 1.0 of the CANOCO package for these calculations (ter Braak & Smilauer 2003). The similarity of assemblage composition among collecting years was estimated with the Morisita-Horn index, comparing abundance data, routinely used in entomology (Magurran & McGill, 2011), and calculated with the R-language function vegdist of the vegan library (Oksanen *et al.* 2011).

Following extensive searches of the BOLD BCISA project, the BOLD public records and GenBank we obtained DNAbarcodes from 58 of the 72 saturniid species recorded in the study for inclusion in our phylogenetic analysis. All data were downloaded from BOLD (including sequences mined from GenBank) either directly or by using the ‘read.BOLD’ function in the R package ‘Spider’ (Brown *et al*., 2012). We took a tree grafting approach to estimating a community level phylogeny. Initially, we generated a well-supported backbone phylogeny using both mitochondrial (COI) and four nuclear (dopamine decarboxylase, wingless, period and carbamoylphosphate synthetase/aspartate transcarbamylase/dihydroorotase) molecular markers (Regier *et al*., 2008). Only taxa for which nuclear sequences were available and for which we could find species level barcodes in the public BOLD database were included. This first analysis consisted of 56 species and 6.4 kb of molecular data. We used the maximum likelihood criterion as implemented in RAxML (Stamatakis, 2006; Stamatakis, Hoover & Rougemont 2008). A gamma model of rate heterogeneity with a proportion of invariant sites was selected and we searched for the best scoring tree after bootstrapping. This best scoring tree was then used as a backbone constraint in a second round of phylogenetic estimation using the same parameters as listed above, but including a dataset of 114 species (including 58 COI taxa from our community sample that were mostly represented by COI only) to be grafted onto the back bone tree. This analysis gave a broader phylogenetic hypothesis, which was checked for obvious mistakes caused by a lack of deeper phylogenetic signal in COI. Obvious incorrect groupings were constrained for a final analysis. We pruned this final tree to include only BCI taxa in subsequent analyses.

##### Saturniid functional attributes and classification

We first used a combination of morphological traits to estimate the ability of a species to disperse (Sekar 2012) and to shift to different habitats in changing environment (Diamond *et al*. 2011; Slade *et al*. 2013). We measured forewing length (FW, mm) and thorax width (TW, mm) with a digital caliper on mounted adult individuals for the most common species and calculated the ratio of TW to FW as an index of flight strength (Wing load). Because of the usually low number of female specimens collected at light traps (Janzen 1984b; Lamarre *et al.* 2015), we measured traits for three male individuals per species. We then build an index of seasonality representing the variance around the peak of maximum abundance of each species per year (hereafter Var_Peak), over the 8 years of monitoring (Lamarre *et al*. 2014). A high variance around the peak of maximum abundance (high Var_Peak) indicates a more generalist occurrence in time and thus a relative low seasonality of adult moths. Alternatively, a low variance around the peak of maximum abundance denotes a narrower peak of abundance and a stronger annual seasonality. For consistency, years during which <4 individuals were recorded were excluded when computing Var_Peak. Note that quarterly surveys within years only allows a low resolution in the estimation of seasonality, but are enough to identify dry or wet season specialists. We also estimated an index of relative host specialization for each taxon (DBIF) as the proportion of host plant family used by the species relative to the total number of host plant families recorded in the database. We compiled Saturniidae host plant records from Janzen & Hallwachs (2016) in Costa Rica (many species are shared between Costa Rica and Panama), from the Hosts database (Robinson *et al*. 2010), from Aiello (2017) in Panama and from unpublished records from Jean-Michel Maes in Nicaragua (total 1,337 host plant records). A small diet index indicates high specialization of the Saturniidae species feeding on fewer host plant families. The overall mean of observations for all species was substituted for missing plant records. The variables FW, TW, Var_Peak and the number of families on which each common species are indicated in Appendix S2.

We were able to quantify functional attributes for 41 species and created a matrix of species x functional traits that included five variables: FW, TW, Wing load, Var_Peak and DBIF. We used this matrix to compute a principal component analysis (PCA). The different components (axes) of the PCA were used as combinations of traits to define a Euclidean functional space with reduced uncorrelated dimensions, where each species was represented according to its PCA scores. This approach is recognized as an efficient way to produce high quality functional spaces where the weight of each synthetic trait is relevant to the fraction of the total trait variance it explains (Villéger, Mason & Mouillot 2008; Maire *et al.* 2015). We then computed a functional tree using hierarchical daisy clustering methods based on functional clusters defined by the species scores on all axes. PCA and clustering approaches were performed using the R packages ade4 (Chessel, Dufour & Thioulouse 2004) and cluster (Maechler *et al.* 2012), respectively; detailed methods for delimitation between the functional groups and validation of the final clustering are provided by Lamarre *et al.* (2016).

Finally, we tested the hypothesis that this trait-based approach represented a functional classification that was independent of moth phylogeny. We mapped each of our clustered functional groups onto the community phylogeny and calculated the mean phylogenetic distance (MPD) between all members of the same cluster. These mean values were compared to randomized distributions as generated by 999 tip label permutations; a two-tailed test was used to assess the probability that mean values fell outside of either tail of the randomized distribution. This test for phylogenetic clustering was conducted as implemented in the R package ‘Picante’ (Kembel *et al*. 2010).

#### Moth population dynamics

To analyze recent population trends, we fitted to each species sufficiently well sampled (i.e., probability to have collected at least one individual per survey each of the 8 study years, total of individuals collected > 32, n = 14 species) three different time-series models (log-linear Poisson regression models) with the software TRIM (Pannekoek & van Strien, 2005), using a matrix of species abundance at each location (n=10) and year (n=8). The program estimates dispersion parameters, and can allow for serial correlation between counts at the same site in different years. The models included: (a) no time-effects: counts vary only across sites and not across time-points; (b) linear trend: model with a site-effect and a linear (on the log-scale) effect of time; and (c) model with separate parameters for each time-point. Goodness of fit of the models was assessed with Chi-square tests and the model with the lowest Akaike information criterion score (AIC) was selected. The base time for these models was Year 2009 (annual index = 1; s.e. = 0). For models linear trend and separate parameters for each time-point, TRIM calculates a multiplicative slope, which corresponds to the yearly change and is converted into one of the following categories, depending on the slope per se as well as its 95% C.L. (Pannekoek & van Strien, 2005):

Strong increase - increase significantly more than 5% per year (5% would mean a doubling in abundance within 15 years). Criterion: lower limit of confidence interval > 1.05.

Moderate increase - significant increase, but not significantly more than 5% per year. Criterion: 1.00 < lower limit of confidence interval < 1.05.

Stable - no significant increase or decline, and it is certain that trends are less than 5% per year. Criterion: confidence interval encloses 1.00 but lower limit > 0.95 and upper limit < 1.05.

Uncertain - no significant increase or decline, but not certain if trends are less than 5% per year. Criterion: confidence interval encloses 1.00 but lower limit < 0.95 or upper limit > 1.05.

Moderate decline - significant decline, but not significantly more than 5% per year. Criterion: 0.95 < upper limit of confidence interval < 1.00.

Steep decline - decline significantly more than 5% per year (5% would mean a halving in abundance within 15 years). Criterion: upper limit of confidence interval < 0.95.

We ran TRIM models for the most common species, for higher taxa together (saturniid family and subfamilies), and for functional groups as delineated by our cluster analysis.

We fitted ARIMA models to our data as explained in the main text. Eventually, we performed a canonical correspondence analyses (CCA) with the software Canoco (ter Braak & Smilauer 2003), to evaluate the influence of climatic variables (Table S1) on the composition of saturniid species within each survey (matrix 41 species x 32 survey; climatic variables selected by stepwise forward selection).

#### References

Aiello, A. & Balcazar, L.M.A. (1997) The immature stages of *Oxytenis modestia*, with comments on the larvae of *Asthenidia* and *Homoeopteryx* (Saturniidae: Oxyteninae). *Journal of the Lepidopterists'* *Society*, **51**, 105-118.

Blest, A.D. (1960b) The evolution, ontogeny and quantitative control of the settling movements of some New World saturniid moths, with some comments on distance communication by honey-bees. *Behaviour*, **16**, 188-253.

Braby, M.F., Eastwood, R. & Murray, N. (2012) The subspecies concept in butterflies: has its application in taxonomy and conservation biology outlived its usefulness? *Biological Journal of the Linnean Society*, **106**, 699-716.

Brechlin, R., Meister, F. & Käch, H. (2014) Vierundzwanzig neue Taxa der Gattung *Oxytenis* Hübner, [1823] (Lepidoptera: Saturniidae, Oxyteninae). *Entomo-Satsphingia*, **7**, 32-57.

Brown, S. D., Collins, R.A., Boyer, S., Lefort, M.C., Malumbres-Olarte, J.A.G.O.B.A*. et al.* (2012). Spider: an R package for the analysis of species identity and evolution, with particular reference to DNA barcoding. *Molecular Ecology Resources*, **12**, 562-565.

Chessel, D., Dufour, A.B. & Thioulouse, J. (2004) The ade4 package-I-One-table methods. *R News*, **4**, 5-10.

Colwell, R.K. (2009) EstimateS: Statistical Estimation of Species Richness and Shared Species from Samples. Version 8.20. User’s Guide and application. University of Connecticut, Storrs. Available at http://purl.oclc.org/estimates.

Dyar, H.G. (1914) Report on the Lepidoptera of the Smithsonian Biological Survey of the Panama Canal Zone. *Proceedings of the United States National Museum*, **47**, 139-350.

Forbes, W.T.M. (1942) The Lepidoptera of Barro Colorado Island, Panama. No. 2*. Bulletin Museum Comparative Zoology*, **90**, 265-406.

Hyams, D.G. (2011) CurveExpert Professional. A Comprehensive Data Analysis Software System for Windows, Mac, and Linux. Version 1.2.2; www.curveexpert.net.

Kembel, S.W., Cowan, P.D., Helmus, M.R., Cornwell, W.K., Morlon, H. *et al*. (2010). Picante: R tools for integrating phylogenies and ecology. *Bioinformatics*, **26**, 1463-1464.

Kitching, R.L., Li, D. & Stork, N.E. (2001) Assessing biodiversity 'sampling packages': how similar are arthropod asemblages in different tropical rainforests? *Biodiversity and Conservation*, **10**, 793-813.

Lamarre, G., Hérault, B., Fine, P. V., Vedel, V., Lupoli, R., Mesones, I., & Baraloto, C. (2016) Taxonomic and functional composition of arthropod assemblages across contrasting Amazonian forests*. Journal of Animal Ecology*, **85**, 227-239.

Lamarre, G. P., Mendoza, I., Fine, P. V., & Baraloto, C. (2014) Leaf synchrony and insect herbivory among tropical tree habitat specialists. *Plant Ecology*, **215**, 209-220.

Leigh Jr, E.G. (1999) *Tropical Forest Ecology: A View from Barro Colorado Island*. Oxford University Press, Oxford.

Lucas, M., Forero, D. & Basset, Y. (2016) Diversity and recent population trends of assassin bugs (Hemiptera: Reduviidae) on Barro Colorado Island, Panama. *Insect Conservation and Diversity*, **9**, 546-558.

Maechler, M., Rousseeuw, P., Struyf, A., Hubert, M., Hornik, K. (2012) Cluster: Cluster Analysis Basics and Extensions. R package version 1.14.2.

Magurran, A.E., & McGill, B.J. (Eds.) (2011) Biological Diversity: Frontiers in Measurement and Assessment. Oxford University Press, Oxford.

Maire, E., Grenouillet, G., Brosse, S. & Villéger, S. (2015) How many dimensions are needed to accurately assess functional diversity? A pragmatic approach for assessing the quality of functional spaces. *Global Ecology and Biogeography*, **24**, 728–740.

Oksanen, J., Guillaume Blanchet, F., Kindt, R. *et al*. (2011) vegan: Community Ecology Package. R package version 1.17-6. Vienna: R Foundation for Statistical Computing. Available at http://CRAN.R-project.org/package=vegan.

Pannekoek, J. & van Strien, A. (2005) TRIM 3 Manual (TRends & Indices for Monitoring data). Statistics Netherlands, Voorburg, Netherlands. Available at <http://www.cbs.nl/en-GB/menu/themas/natuur-milieu/methoden/trim/default.htm?Languageswitch=on>

Regier, J.C., Grant, M.C., Mitter, C., Cook, C.P., Peigler, R.S. & Rougerie, R. (2008) Phylogenetic relationships of wild silkmoths (Lepidoptera: Saturniidae) inferred from four protein-coding nuclear genes. *Systematic Entomology*, **33**, 219-228.

Robinson, G.S., Ackery, P.R., Kitching, I.J., Beccaloni, G.W. & Hernández, L.M. (2010) HOSTS - A Database of the World's Lepidopteran Hostplants. Natural History Museum, London.

Rougerie, R. (2008) Lepidoptera Barcode of Life: Saturniidae. Available at <http://www.lepbarcoding.org/saturnidae/species_checklists.php>.

Stamatakis, A. (2006) RAxML-VI-HPC: maximum likelihood-based phylogenetic analyses with thousands of taxa and mixed models. *Bioinformatics*, **22**, 2688.

Stamatakis, A., Hoover, P. & Rougemont, J. (2008) A rapid bootstrap algorithm for the RAxML web servers. *Systematic biology*, **57**, 758-771.

ter Braak, C.J.F., Smilauer, P. (2003) CANOCO Reference Manual and User’s Guide to Canoco for Windows: Software for Canonical Community Ordination (version 4.52). Microcomputer Power, Ithaca, NY, USA.

Valtonen,, A., Molleman, F., Chapman, C.A., Carey,J.R., Ayres, M.P. , Roininen, H. (2013) Tropical phenology: bi-annual rhythms and interannual variation in an Afrotropical butterfly assemblage. *Ecosphere*, **4**,:36. http://dx.doi.org/10.1890/ES12-00338.1

Villéger, S., Mason, N.W., & Mouillot, D. (2008) New multidimensional functional diversity indices for a multifaceted framework in functional ecology. *Ecology*, **89**, 2290-2301.

**Table S1.** Climatic variables considered in regression analyses and CCA. Apart from the ENSO index, variables are available at http://biogeodb.stri.si.edu/physical_monitoring/research/barrocolorado. The codes for CCA refer to Fig. S6.

| **Variable** | **Description (units)** | **Code for CCA** |
| --- | --- | --- |
| Related to survey |  |  |
| AvTmax | Average maximal daily temperature during the 4 days of the survey (°C) | AvTmax |
| AvTmin | Average minimal daily temperature during the 4 days of the survey (°C) | - |
| SumRainfall | Sum of rainfall during the 4 days of the survey (mm) | - |
| AvMoon | Mean percentage of moonlight during the 4 days of the survey (%) | Moon |
|  |  |  |
| Related to month |  |  |
| ENSO* | Monthly El Niño / Southern Oscillation (ENSO) Index, available at:  http://www.cpc.ncep.noaa.gov/products/analysis_monitoring/ensostuff/ensoyears.shtml | - |
| SoilHumMonth | Average soil humidity during the month of the survey (%) | SoilHumidity |
| SolarRadMonth | Average solar radiation flux during the month of the survey (MJ m-2 day-1) | - |
| TmaxMonth | Average maximal daily temperature during the month of the survey (°C) | - |
| TminMonth | Average minimal daily temperature during the month of the survey (°C) | - |
| SumRainfallMonth | Sum of rainfall during the month of the survey (mm) | RainMonth |
|  |  |  |
| Time lags |  |  |
| AvTmax15 | Average maximal daily temperature during the 15 days before the survey (°C) | - |
| AvTmax30 | Average maximal daily temperature during the 30 days before the survey (°C) | - |
| AvTmin15 | Average minimal daily temperature during the 15 days before the survey (°C) | AvTmin15 |
| AvTmin30 | Average minimal daily temperature during the 30 days before the survey (°C) | - |
| DegreeDays15** | Sum of degree-days for the 15 days preceding the survey (°C) | - |
| DegreeDays30** | Sum of degree-days for the 30 days preceding the survey (°C) | DegreeDay30 |
| SumRain15 | Sum of rainfall during the 15 days before the survey (mm) | - |
| SumRain30 | Sum of rainfall during the 30 days before the survey (mm) | - |

* Years 2011, 2015 and 2016 are qualified as weak to moderate La Niña event, very strong El Niño event and very strong El Niño event, respectively (National Oceanographic and Atmospheric Administration, 2016).

**Calculated as Degree-days = (Maximum temperature + Minimum temperature/2) - Minimum threshold, with Minimum threshold set to 18°C (i.e., minimum temperature at night in the tropics; Arnold, 1960).

Arnold, C.Y., 1960. Maximum and minimum temperatures as a basis for computing heat units. Proc. Am. Soc. Hortic. Sci. 74, 430–445.

National Oceanographic and Atmospheric Administration. 2016. <http://www.cpc.ncep.noaa.gov/products/analysis_monitoring/ensostuff/ensoyears.shtml>

**Table S2.** Summary of the density and quality of molecular data, for the Saturniidae recently collected on Barro Colorado Island. Means are displayed ± s.e.

| **Variable** | **Value** |
| --- | --- |
| Number of species / Number of different BINs | 33 / 33 |
| Total number of sequences | 167 |
| Average number of sequences per species (Min - Max) | 5.1 ± 0.55 (1 - 11) |
| Average sequence length (Min - Max) | 655.8 ± 0.73 base-pairs (599 – 658) |
| Average normalized divergence within species (Min - Max) | 0.092 ± 0.008 % (0 – 0.93) |
| Average normalized divergence within genus (Min - Max) | 9.5 ± 0.057 % (4.6 – 14.5) |
| Average distance to nearest neighbor (Min - Max) within the dataset | 9.6 ± 0.457 % (4.8 – 13.9) |

**Table S3.** Lower matrix of faunal similarity (Morisita-Horn index) among collecting years, 2009-2016.

| **Year** | **2009** | **2010** | **2011** | **2012** | **2013** | **2014** | **2015** |
| --- | --- | --- | --- | --- | --- | --- | --- |
| 2010 | 0.645 | - | - | - | - | - | - |
| 2011 | 0.785 | 0.674 | - | - | - | - | - |
| 2012 | 0.742 | 0.840 | 0.721 | - | - | - | - |
| 2013 | 0.678 | 0.659 | 0.454 | 0.649 | - | - | - |
| 2014 | 0.577 | 0.596 | 0.514 | 0.534 | 0.621 | - | - |
| 2015 | 0.669 | 0.522 | 0.470 | 0.690 | 0.615 | 0.255 | - |
| 2016 | 0.741 | 0.636 | 0.599 | 0.718 | 0.767 | 0.679 | 0.701 |

**Table S4.** Main functional trends for each clustered functional groups and main results of the TRIM time-series and phylogenetic signals. Results = actual trends in the present study. Observed mean phylogenetic distance (MPD) between members of the same functional group, the randomized mean (generated using 999 tip label swaps) and significance (two-tailed test) given in the last three columns.

| **Cluster**  **#** | **Dispersal** | **Annual** | **Host plant** | | **Dominant** | **Functional trends** | **Results**  **(TRIM)** | **Observed MPD** | **Random MPD** | ***p*** |
| --- | --- | --- | --- | --- | --- | --- | --- | --- | --- | --- |
|  |  | **seasonality** | | **Specialization** | **taxa** |  |  |  |  |  |
| 1 | High | High | High | | All Ceratocampinae | Large species – Host specialist - Seasonal | TRIM not significant | 0.537 | 0.686 | **0.066** |
| 2 | Low | Low | Low | | Only Hemileucinae (*Automeris*) | Small to medium – Host generalist - Aseasonal | **TRIM uncertain** | 0.377 | 0.688 | **0.005** |
| 3 | Low | Low | High | | All Oxyteninae and Hemileucinae (*Automeris*) | Small to medium– Host specialist - Aseasonal | TRIM not significant | 0.705 | 0.687 | 0.612 |
| 4 | Low | Low | Low | | Mostly Hemileucinae (*Hylesia*) and two Ceratocampinae species | Small to medium – Host generalist - Aseasonal | TRIM not significant | 0.610 | 0.687 | 0.154 |
| 5 | High | High | High | | All Arsenurinae | Large species – Host specialist – Seasonal | **TRIM moderate increase** | 0.428 | 0.688 | **0.002** |

**Table S5.** Summary results of stepwise multiple regressions with abundance as dependent variable and climatic variables as independent variables (coded as in Table S1). Variables are listed in decreasing order of absolute value of standard coefficients. Variables in red have a positive coefficient; variables in blue have a negative coefficient.

| **Taxa/Group** | **Significant variables** | **F** | **p** | **R2** |
| --- | --- | --- | --- | --- |
| All Saturniidae | AvTmin, AvTmax15, SumRainfall, SolarRadMonth, AvTmin30 | 5.47 | 0.001 | 0.419 |
| Arsenurinae | DegreeDays30, AvTmin30, DegreeDays15, TminMonth, ENSO, SolarRadMonth, SumRainfall | 6.90 | 0.001 | 0.571 |
| Ceratocampinae | DegreeDays30, DegreeDays15, AvTmax30, AvTmax15, AvTmin30, AvTmin15, SumRainfall, AvTmin, AvTmax | 7.69 | 0.001 | 0.660 |
| Hemileucinae | AvTmin30, AvTmin, AvTmax, SumRainfall, SoilHumMonth | 4.58 | 0.004 | 0.366 |
| Oxyteninae | SoilHumMonth, SumRainfallMonth, DegreeDays30, SumRain15 | 6.89 | 0.001 | 0.432 |
|  |  |  |  |  |
| Functional group 1 | DegreeDays30, DegreeDays15, AvTmax15, AvTmax30, AvTmin30, SolarRadMonth, SoilHumMonth, | 8.57 | 0.001 | 0.745 |
|  | TminMonth, AvTmax, SumRainfallMonth, ENSO, AvMoon |  |  |  |
| Functional group 2 | SoilHumMonth, SumRain30, SumRainfallMonth, TmaxMonth | 9.91 | 0.001 | 0.535 |
| Functional group 3 | DegreeDays30, DegreeDays15, AvTmax30, AvTmin30, AvTmax15, AvTmin15, SoilHumMonth, TmaxMonth, | 5.37 | 0.001 | 0.585 |
|  | AvTmin, SumRain15 |  |  |  |
| Functional group 4 | DegreeDays30, DegreeDays15, AvMoon | 3.51 | 0.028 | 0.195 |
| Functional group 5 | DegreeDays30, AvTmin30, SumRainfall, AvMoon, SolarRadMonth | 11.99 | 0.001 | 0.639 |
|  |  |  |  |  |
| *Rhescyntis hippodamia* | DegreeDays30, AvTmin30, SumRainfall | 10.17 | 0.001 | 0.470 |
| *Hylesia praedpichinchensis* | DegreeDays30, DegreeDays15, AvMoon | 4.00 | 0.017 | 0.225 |
| *Automeris parapichinchensis* | AvTmax15, SoilHumMonth, DegreeDays15 | 5.78 | 0.003 | 0.316 |
| *Automeris vanschaycki* | SumRainfallMonth, TmaxMonth, SumRainfall, AvMoon, AvTmin30 | 9.60 | 0.001 | 0.581 |
| *Periphoba* sp. 1YB | DegreeDays30, DegreeDays15, SumRain15, AvTmin30, TminMonth, SumRain30, | 4.12 | 0.003 | 0.475 |
|  | SumRainfallMonth, AvTmax, SumRainfall |  |  |  |
| *Citheronia lobesis* | DegreeDays30, AvTmax30, AvTmax15, AvTmin30, DegreeDays15, SumRain30, SolarRadMonth, SumRain15, | 6.83 | 0.001 | 0.674 |
|  | SumRainfallMonth, AvTmax, AvMoon |  |  |  |
| *Automeris fieldi* | DegreeDays30, DegreeDays15, AvTmax30, AvTmax15, AvTmin30, TminMonth, SoilHumMonth, AvMoon | 3.50 | 0.009 | 0.392 |
| *Pseudodirphia eupanamensis* | SumRainfall, AvTmin30, AvTmax15, AvTmin | 5.45 | 0.002 | 0.364 |
| *Pseudautomeris salmonea* | DegreeDays15, AvTmax15, AvTmin, ENSO | 4.60 | 0.006 | 0.317 |
| *Oxytenis naemia* | AvTmax15, AvTmax30, SumRainfallMonth, SoilHumMonth, TmaxMonth | 3.74 | 0.011 | 0.306 |
| *Automeris belti* | SumRainfallMonth, AvTmax30, TmaxMonth, AvTmax15, TminMonth, AvMoon, AvTmin | 4.55 | 0.002 | 0.445 |
| *Caio championi* | DegreeDays30, SumRain30, AvTmax, SumRain15, AvTmin15, SolarRadMonth | 7.38 | 0.001 | 0.553 |
| *Titaea tamerlan* | DegreeDays30, DegreeDays15, AvTmax30, AvTmin30, AvTmax15, AvTmin15, AvTmin, SumRainfall, AvMoon | 5.19 | 0.001 | 0.549 |
| *Oxytenis beprea* | AvTmax15, DegreeDays15, SoilHumMonth, SumRainfallMonth, AvTmin, SumRain15 | 2.80 | 0.032 | 0.259 |

**Fig. S1.** Lowess smoothing of annual maximum temperature (monthly mean; red), annual minimum temperature (monthly mean, blue) and annual rainfall (sum) during the study period (2009-2016).

**Fig. S2.** (a) Mean cumulative number of species collected during the period 2008-2016 plotted against the cumulative number of individuals collected with ForestGEO light traps. Grey lines are 95% C.L. (b) Cumulative no. of individuals sequenced plotted against the cumulative no. of cryptic species discovered, for years 2008-2012. The grey line represents the best fit model, with its equation in inset.


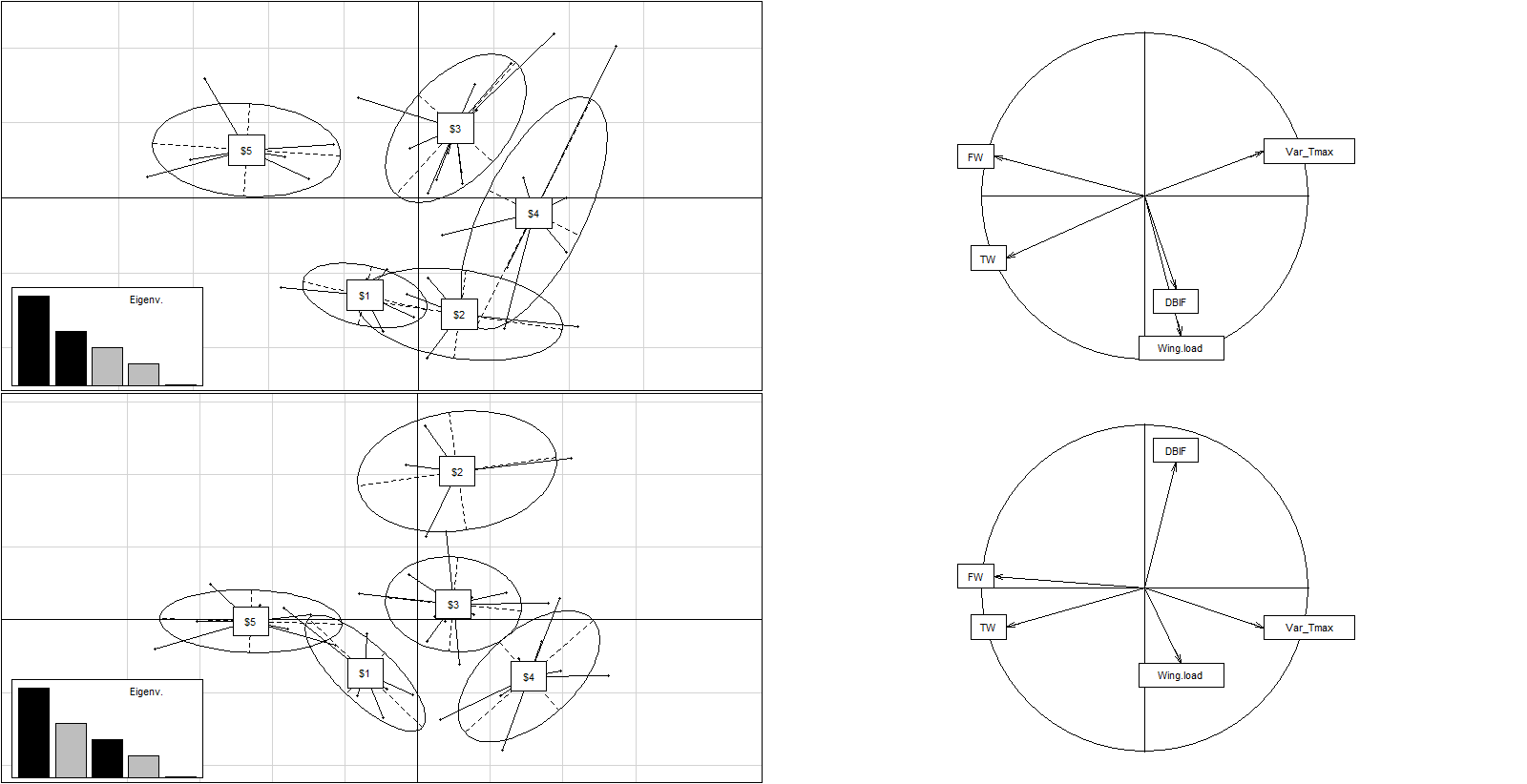


Axis 2

Axis 3

Axis 1

Var_Peak

Var_Peak

**Fig. S3**. Results from the PCA showing the location of the clustered functional groups (cluster S1, S2, S3, S4 and S5) in the Euclidian space analyzed using the five functional attributes (functional trends as described in Table S4). Overall, Axis 1 explains 22% of the variation in the illustrated functional space while Axes 2 and 3 explained 13% and 9%, respectively. The five functional traits (FW, TW, Wing load, Var_Peak and DBIF) accounting for dispersal ability, seasonality and host specialization of saturniid moths are plotted separately in correlation circles to show the load and contribution of each functional trait on each axis of the PCA.

**Figure S4**. The saturniid community phylogeny for BCI with the functional clusters (1-5) of each taxon marked with black circles at the tips.

Axis 3

**Fig. S5.** Population changes on BCI during 2009-2016 for higher taxa, functional groups and species not detailed in Fig. 5. Grey lines: raw data; black lines: fitted TRIM models [±SD] (Table 2); dashed orange lines: fitted regressions (Table S5); dashed red lines: forecast of ARIMA models for 2017-2018; and dashed green lines: 95% confidence limits of ARIMA models.

**Fig. S6.** Plots of surveys and climatic variables in Axes 1,2 of the CCA constraining 41 species of saturniids across 32 surveys. Surveys are coded as M = March, Y = May, S = September, N = November and the two last digits identifying the year (2009-2016). Climatic variables coded as in Table S1.

**Appendix S2.** List of species of Saturniidae collected or observed on Barro Colorado Island, for the period 1958-2016. The number of specimens is summed for whole study period (Abundance), and detailed for each year on record. When available, Barcode Index Numbers (BINs), sequences included in the phylogenetic tree and their length (bp) are also indicated for each species.

**Appendix S3.** Supporting data for reproducing the phylogenetic tree in Fig.2, including a Newick format tree file.
