# Supplementary material for: The Saturniidae of Barro Colorado Island, Panama: A model taxon for studying the long‐term effects of climate change?
Source: Ecol Evol. 2017 Oct 22;7(23):9991–10004. doi: 10.1002/ece3.3515 (PMC5723595; doi:10.1002/ece3.3515)
Supplement: Supplementary file 3 [file ECE3-7-9991-s003.doc]

((Therinia_t:0.1593881874,(Homoeopter:0.183304908,((Oxytenis_3:0.1650778388,Oxytenis_2:0.1432295503):0.02681794745,(Oxytenis_4:0.09762208121,Oxytenis1C:0.1115141113):0.02967522778):0.1070279537):0.05295072785):0.1088664314,(((Rothschil2:0.194191775,Rothschil1:0.1036119482):0.1033487983,Copaxa_dec:0.1871450102):0.09569208902,((((((Adeloneiv2:0.1792742636,Adeloneiv1:0.2529934708):0.07271434463,Ptiloscola:0.2288970858):0.03961899021,(Othorene_p:0.2433995948,(Syssphinx1:0.1883135434,Syssphinx2:0.1758131083):0.03598709787):0.05897555254):0.06417146205,((Schausiell:0.1517724301,(Eacles_im2:0.1697807567,Eacles_pen:0.1421832804):0.04654754384):0.03288416068,Citheroni2:0.1685638868):0.01098714863):0.07641908368,((((Periphoba_:0.2466748146,((Pseudodir2:0.1600553788,Pseudodir3:0.1407427958):0.07944481926,(Pseudodir1:0.1696372635,Pseudodirp:0.07078684861):0.01670878833):0.11479563):0.04497358111,(Cerodirphi:0.3914262325,(Molippa_la:0.09482240627,Molippa_si:0.08567003939):0.0565028011):0.02131925791):0.005708884885,((((Automeri12:0.07104813495,Automeris8:0.05590558282):0.1594154965,(Automeris7:0.1505587516,(((Automeris3:0.08396276076,(Automeri11:0.1667282038,Automeris5:0.09027882892):0.01057066031):0.03374960048,(Automeri10:0.1040317783,(Automeris9:0.07044620261,Automeris4:0.07166718125):0.008756349404):0.01873950788):0.02013355658,(Automeris2:0.03888873381,Automeris6:0.02432607679):0.121484741):0.006836206411):0.01269539639):0.003302154257,Pseudautom:0.1126437363):0.01490704685,((Hylesia_co:0.1152515862,((Hylesia_um:0.04191074224,(Hylesia_ru:0.04234725503,(Hylesia_p2:0.03818642854,Hylesia_p1:0.02293915892):0.03775285237):0.0145263252):0.01085741712,(Hylesia_ae:0.1323599492,(Hylesia_da:0.07583968674,Hylesiaann:0.08833180679):0.007480127121):0.0164829276):0.02093638276):0.0785885272,(Gamelia_sp:0.165304256,(Automerina:0.2415889954,Hyperchiri:0.2380484206):0.05710591659):0.01871565201):3.376844787e-06):0.02042106426):0.05942425571,(Lonomia_el:0.2295268874,Periga_cyn:0.2794234255):0.02627762382):0.1077306398):0.01602279051,(((Caio_champ:0.2076455674,(Titaea_ta2:0.09987273364,Dysdaemon2:0.07260951297):0.0351915153):0.01800554879,(Copiopter2:0.1680047339,Rhescynti2:0.1114016538):0.01027348792):0.006632112859,Arsenura_b:0.2394001267):0.05932652472):0.02173912429):0.1348204762);
